# Canadian copyright protections for neurodata: ethical and legal implications

**DOI:** 10.1093/jlb/lsag008

**Published:** 2026-03-14

**Authors:** Kyrstin Lavelle, Reina Magistro Nadler, Margot Gunning, Graham J Reynolds, Judy Illes

**Affiliations:** Neuroethics Canada, Division of Neurology, Department of Medicine, University of British Columbia, Vancouver, BC, Canada; Neuroethics Canada, Division of Neurology, Department of Medicine, University of British Columbia, Vancouver, BC, Canada; Allard School of Law, University of British Columbia, Vancouver, BC, Canada; Neuroethics Canada, Division of Neurology, Department of Medicine, University of British Columbia, Vancouver, BC, Canada; Allard School of Law, University of British Columbia, Vancouver, BC, Canada; Allard School of Law, University of British Columbia, Vancouver, BC, Canada; Neuroethics Canada, Division of Neurology, Department of Medicine, University of British Columbia, Vancouver, BC, Canada

**Keywords:** neurodata, copyright, data governance, moral rights, neuroethics

## Abstract

This essay examines how Canadian copyright law treats neurodata generated for neuroprediction and further probes if copyright or similar protections would offer mechanisms to safeguard individuals who produce those data. Using a hypothetical fact pattern, we apply the conditions for subsistence of copyright to neurodata created by a research participant and processed by a researcher. The results of the analysis indicate that both parties can credibly argue that copyright subsists in the neurodata, although such an outcome is neither established nor guaranteed under current law. We then explore the policy significance of this legal analysis from a neuroethics perspective. Drawing together literatures on data justice, political economy, and neurotechnology governance, we argue that when people produce neurodata, legal systems should appropriately honor their contributions. This could be accomplished through protections of the integrity of neurodata from harmful misuse, akin to what moral rightsholders can accomplish under Canadian moral rights doctrine. We further highlight the need to protect individual autonomy over brain data, whether via copyright or another mechanism. We conclude that the Canadian approach to copyright law and moral rights offers a model for policy and governance as neurodata find their way into legally and socially consequential technologies.

## I. INTRODUCTION

This essay explores legal and ethical dimensions of Canadian copyright law as they pertain to neurodata generated for research. We first investigate whether neurodata produced in a neuroprediction research study would be copyrightable under the Canadian *Copyright Act*. We carry out the copyright analysis as applied both to a research participant and a researcher since each contributes to the production of neurodata in a distinct way. We find that each of these parties can make plausible arguments for copyright protection in the data. Turning to ethical and policy considerations, we discuss the types of protection that society owes to producers of neurodata, whether under copyright law or other frameworks. The neuroethics analysis offers an appraisal of policy tensions and human stakes attendant to this convergence of emerging neurotechnology and intellectual property, and outlines a way forward for policymakers to empower research participants—data subjects—against harmful misuse of their own neurodata. While the legal conclusions drawn here apply specifically to Canada—a jurisdiction with an upsurging neurotechnology sector and robust moral rights protections—the recommendations can be adapted globally.

### I.A. Neurotechnology, Neurodata, and Neuroprediction

Neurotechnology broadly encompasses devices that combine aspects of neuroscience, engineering, and technology to generate information or influence the function of the brain or nervous system.^1^ Neurotechnological innovation is quickly proliferating. Between 2000 and 2020, patent offices around the world saw a 20-fold increase in neurotechnology patent applications.^2^ The capabilities realized by neurotechnology span multiple industries, with applications ranging from healthcare to marketing.^3^ The increased interest and investment in this technology are driven by perceived scientific, medical, and economic benefits.^4^ As neurotechnology becomes more sophisticated, concerns regarding ownership, governance, and ethical use of the data it generates are growing.^5^ Unlike more familiar forms of personal health data (eg cardiac data), neurodata are derived directly from the brain and are especially personal.^6^ Neurodata bear intimate connections to the data subject, enabling a potentially exceptional depth of insight into personal brain function and mental states.^7^ While many forms of biometric or medical data are similarly revealing, neurodata combine significant inferential power with person-specific uniqueness rivaling that of a fingerprint. The data often bear hallmarks not only of individuality and identity, but of a person’s voluntary creative effort. The lack of consistent approaches to ownership and control of neurodata across jurisdictions may leave individuals on legally uncertain footing, vulnerable to unwanted exposure.^8^

These concerns become salient in relation to neuroprediction, the capability to make predictions from information—neurodata—produced by the brain or nervous system.^9^ Such neurodata have a variety of applications in research contexts, including generation of predictive inferences regarding memory content, mood, and aptitude.^10^ Investigators have used neurotechnology, for example, to predict treatment responses for major depressive disorder from measures of cortical thickness (a structural variable), amygdala activity (a functional variable based on blood flow and regional oxygenation levels), and synaptic connectivity (a neurophysiological variable).^11^ Neuroprediction can also enable behavioral forecasting in certain contexts. For example, neuroimaging, clinical psychiatric evaluations, brain age estimation, and behavioral marker studies using both univariate and multivariate statistical approaches have accurately predicted an increased likelihood of recidivism.^12^ In this regard, commentators widely consider neuroprediction technologies to hold significant potential to influence decisions in settings ranging from healthcare to law.^13^

Algorithms play an increasingly significant role in processing neurodata. Machine learning (ML) is a programing approach where the behavior of programs can be generative, capable of producing new information and of analyzing brain function to extract meaningful patterns.^14^ For example, ML can be used to enhance image characterization or the prediction of outcomes by extracting and analyzing complex neural patterns.^15^ Deep learning, a variant of ML, can transform raw data by using a set of algorithms that progressively produce more complex and abstract patterns in large datasets.^16^ Deep learning techniques such as convolutional neural networks are used in neuroimaging for feature extraction, while recurrent neural networks and reinforcement learning models support dynamic brain activity predictions. These types of algorithmic prediction methods are growing in prevalence, with some techniques capable of inferring some mental states from brain activity or categorizing individuals based on neurobiological features such as brain structure.^17^ The quality of the prediction depends on factors including the specifics of human instruction and any biases in the neurodata sets on which the algorithm is trained.

Throughout this discussion, we will refer to neurodata in two ways: raw data and outcome data. These should not be understood as establishing a strict dichotomy, but rather as highlighting earlier and later stages of data processing. Thus, the phrase ‘raw data’ here connotes neurodata that have been collected without undergoing further computational transformation or statistical analysis. The phrase ‘outcome data’ connotes downstream neurodata, after the researcher has applied whatever extent of skill and judgment the process demands of them. Owing to the growing role of ML algorithms in neuroprediction, many scenarios, including our hypothetical below, will involve integrating neurodata with a neuropredictive ML algorithm. The type and extent of algorithmic processing applied to turn raw data into outcome data may impinge on certain prongs of the copyright analysis below.

### I.B. The Role of Canadian Copyright

Neurodata frequently embed personal traits about individuals exceeding what is embedded in other data types, making governance particularly complex.^18^ Separate from any purpose-built regulatory frameworks that might apply to neurodata such as privacy or health legislation, we consider the potential for copyright law to effectively add a layer of data governance. We therefore begin by addressing whether copyright can subsist in neurodata in the first place.

In Canada, copyright law is uniformly governed across all provinces and territories by the *Copyright Act (R.S.C., 1985, c. C-42 [Copyright Act]).*^19^ The *Copyright Act* grants exclusive rights to creators in their works of expression, including literary, dramatic, musical, and artistic works.^20^ Three landmark Canadian cases have defined the purpose of copyright to both reward owners and creators, and encourage public dissemination of and access to intellectual works.^21^ In order to qualify for copyright, a work must meet several requirements in statutory and common law. Different commentators parcel out these requirements in different ways. Our gloss on the copyright test is five-part: nexus, expression, fixation, originality, and work.^22^

The Canadian *Copyright Act* does not explicitly address neurodata. The unique characteristics of data derived from the brain may strain the applicable legal concepts, which reflect assumptions about human creativity and expression that predate neurotechnology.^23^ As technology advances and neuroprediction gains prominence, addressing these challenges and conceptual gaps is vital. To that end, we ask two central questions:

1. **Legal Analysis:** Would individual producers of neurodata, whether researchers or research participants, be able to successfully claim protection of their data under Canada’s *Copyright Act*?
2. **Ethical Implications:** If copyright may indeed subsist in neurodata under Canadian law, how might the result inform the best approach for upholding neurodata producers’ rights and interests, such as privacy and autonomy?

The legal analysis is intended to inform the ethics and policy discussion. We apply the relevant tests in copyright law not only to understand possible outcomes if this issue is someday litigated, but to highlight normatively important aspects of neurodata creation. Placing neuroethics themes and principles into conversation with growing literatures at the intersection of these two questions, we argue that neurodata producers deserve a policy system offering rights commensurate with the personal nature of these data types. Those rights entail some ability to object, within limitations, to harmful or prejudicial subsequent use. While copyright might function adequately in this regard, an expanded purpose-built policy framework would offer better protections overall. In the future, we hope to engage in research detailing what such a framework might look like.

## II. APPROACH

We use the example of recidivism prediction to focus the legal analysis. Research demonstrates that it is possible to predict recidivism—criminal reoffending—by using a single neurobiological variable or, more robustly, by collecting and combining a range of factors (eg demographic information, delinquency and substance use history, behavioral traits, and neurobiological markers) into a multimodal predictive model.^24^ Researchers collect neurodata and other forms of prediction-relevant data using various methods. Certain assessments of behavioral variables, such as aggression, are still collected via simple self-report instruments.^25^ Autonomic nervous system data, including heart rate, can be captured using tools such as ambulatory monitoring systems.^26^ Neurodata gathered directly from the brain include evoked potentials, which can be measured using electroencephalography (EEG). These can reveal error processing while participants perform exercises that measure cognitive control, such as the Eriksen flanker task.^27^ Studies have also evaluated behavioral impulsivity through functional magnetic resonance imaging (fMRI) during go/no-go tasks, which require participants to inhibit a prepotent motor response.^28^ The datasets compiled from multimodal assessments (behavioral, autonomic, electrophysiological, and neuroimaging) are then integrated into an algorithmic predictive model.

We consider these capabilities in the hypothetical case of a person imprisoned for a crime of aggression—Melville Olivier—and researcher Dr Liv Maple, MD PhD:

Melville Olivier is a 27-year-old white male Canadian citizen and an inmate at a prison near the city of Winnipeg in the Canadian province of Manitoba. While in the second year of serving his eight-year sentence for a violent robbery, Olivier was offered the opportunity to participate in a research study on recidivism neuroprediction. There were no incentives or inducements to participate, but alongside the informed consent documentation there was a waiver of all economic rights to any intellectual property (IP) generated in the course of the study. The waiver, a precondition for study participation, made no mention of other kinds of rights. Olivier, motivated to participate, voluntarily signed the waiver*.*

Olivier was asked to complete self-reported surveys relating to predictive variables including demographics, sex, and age. He then received training in two brain exercises that he would need to perform while being monitored: the Eriksen flanker task to measure error processing, and a go/no-go task in a mobile fMRI unit as a metric of impulsivity. Olivier’s resulting dataset—a compilation of his neurobiological, autonomic, behavioral, and self-report data for use in the creation of a multimodal algorithmic predictive model—was saved to the research team’s hard drive*.*

The lead researcher, Dr. Maple, is a Canadian citizen, employed by a self-standing non-profit research institute in Winnipeg. The standard terms of employment for faculty and researchers assign economic IP rights created in the course of their work to the institute, with no waivers for other kinds of rights. Dr. Maple reviewed and interpreted datasets from Olivier and the other participants with the partial assistance of specialized software powered by a machine learning algorithm. She applied her expertise to make adjustments to the parameters of the data analysis and ultimately produced a set of predictive scores regarding the likelihood that Olivier and others in the study will reoffend*.*

Two years after the research is conducted, Olivier and Dr. Maple (who has moved to a new job) each independently discover an investigative journalist’s article on neuroprediction technologies. It reports that neurodata from several research studies, including the one conducted by Dr. Maple, were licensed to an AI startup for the purpose of training a commercially available recidivism neuroprediction algorithm. The article notes that the developer of the algorithmic tool later contracted with the Correctional Service of Canada (CSC) to help the agency implement a neuroprediction system in prisons. The journalist notes that CSC’s new neuropredictive approach has coincided with an increase in overall sentence lengths served, but declines to speculate whether the relationship is causal*.*

Olivier, who is still in prison for another four years, and Dr. Maple each wonder what legal rights they each have with respect to the neurodata collected in the original research study*.*

Our treatment of this hypothetical case suggests that there are good reasons to believe copyright subsists in the neurodata as described. Moreover, it will demonstrate that both the researcher and participant have undertaken expressive activities that reflect their individuality. Copyright law recognizes the inherent value of such activities, and in doing so it offers a lesson worth incorporating into neuroethical and policy discourse.

## III. COPYRIGHT ANALYSIS

The purpose of the Canadian *Copyright Act*, as stated by Justice Binnie writing for the majority in *Théberge v. Galerie d’Art du Petit Champlain Inc.*, 2002 SCC 34, is to balance ‘… promoting the public interest in the encouragement and dissemination of works of the arts and intellect’ against ‘obtaining a just reward for the creator’.^29^ Copyright automatically subsists where works meet the criteria that are defined by the *Act* and refined in case law. The *Act* and relevant case law lay out five elements for works to be copyrightable: nexus, expression, fixation, originality, and work. We next apply these five elements to the hypothetical case.

### III.A. Nexus Requirement

Section 5(1) of the *Copyright Act* sets out the conditions under which copyright subsists in Canada. The relevant condition in our case, § 5(1)(a), provides that copyright will subsist where authors of copyrightable works are citizens or subjects of—or persons ordinarily resident in—any treaty country as defined in the Act, which includes Canada.^30^ Nexus is thus a requirement for copyright in Canadian cases, and is satisfied explicitly for both individuals, as both were Canadian citizens and residents during the research study.

### III.B. Expression Requirement

In Canada, copyright is only available for the expression of an idea, not for the idea itself. This dichotomy between ideas and their expressions underpins the expression requirement, first established in *Kenrick v. Lawrence* (1890) LR 25, QBD 99 [*Kenrick*]. The *Kenrick* court held that a work must be embodied beyond a mere mechanical exercise to count as expressive.^31^ This principle was reaffirmed in *Winkler v. Hendley,* (2021) FC 498 [*Winkler*], in which the Federal Court reiterated that copyright does not protect ideas or facts, only their particular expression.^32^ In many cases, courts can negotiate this distinction straightforwardly by asking whether the work represents a particular, distinctive instantiation of an idea, or simply relays a pure idea in a more general and unspecified manner.

The idea/expression dichotomy, as well as the usual focus on ideas themselves, may become complicated in the context of neurodata. Under circumstances where a person’s brain activity is monitored, the very formation of an idea arguably doubles as a particularized expression of that idea. The most in-depth exploration of this complexity comes from Dutch lawyer Radha Pull ter Gunne, who considered it in the context of Dutch and EU copyright law.^33^ Pull ter Gunne questioned if brain data recorded via a brain computer interface (BCI) should be considered a copyright-eligible expression or merely an uncopyrightable idea, noting that BCIs effectively expand the ‘grey area where the idea and the expression are intertwined’.^34^ In such gray areas, it is no longer helpful to ask whether the idea is general or specific: those descriptors map poorly onto a pre-verbal, inchoate thought embodied in unique networks of interacting neurons. In the Canadian fact pattern, the expression analysis unfolds in this gray area, bolstering Olivier’s case for copyright.

#### 1. As Applied—Research Participant

Olivier’s dataset uniquely represents his mental processes, reflecting his brain’s active, voluntarily mediated enactment of ideas. In this way, the translation of thoughts to recorded neurodata could be analogous to song writing or recording, a familiarly copyrightable type of expressive work.^35^ Much like an artist putting lyrics to paper or directing voice into a recorder, Olivier placed his thoughts within the detection aperture of the scanner and electrodes.

This case therefore diverges from the situation recounted in *Andrews v. McHale*, 2016 FC 624 [*Andrews*]. In *Andrews*, the Federal Court of Canada addressed whether the plaintiff’s non-coding contributions to four software programs could qualify as copyrightable joint authorship, though he did not write the actual code. The court concluded that his contributions were in the category of ‘ideas, methods, procedures, algorithms or other categories of contributions which, while perhaps valuable, fall outside the type of intellectual effort protected by copyright law …’.^36^

In a scenario where Olivier had passively allowed for the capture of only structural neurodata and nothing else, he might fail the expression requirement for lack of intellectual effort. Here, however, the data collected during the Eriksen flanker and go/no-go tasks are functional in nature^37^; while Olivier did not externalize his responses via utterance, he nonetheless engaged in an expressive process. Olivier’s raw neurodata set satisfies the expression requirement because it is the concrete, non-abstract result of voluntary ‘intellectual effort’—a key missing factor in the *Andrews* case.^38^ It would amount to a mischaracterization of the basic neuroscience and type of neurodata at issue in this case to frame the dataset itself as somehow a collection of abstract, unexpressed ideas.^39^

#### 2. As Applied—Researcher

Dr Maple’s efforts in compiling and analyzing the dataset, and especially in producing the predictive outcome data, likely satisfy the expression requirement. Following the precedent set by *Kenrick* and further elaborated in *Winkler*, an original expression of an idea is protected.^40^ Dr Maple engaged in human interpretation and creative presentation, transforming raw information into an original, tangible form. The process involved selection, organization, and interpretation of data beyond simple mechanical recording.^41^

### III.C. Fixation Requirement

The fixation requirement, as developed in common law, demands that a copyrightable work exist in a tangible and fixed form.^42^ One way to do this is by saving data to a hard drive.^43^ With respect to both Olivier and Dr Maple, the fixation requirement is uncontroversially satisfied: the data generated in each step were digitally recorded.

### III.D. Work Requirement

Copyright protections can only apply to products that the *Copyright Act* defines either as a ‘work’ or as one of a few other forms of copyrightable subject matter not relevant here. Per section five of the law, ‘copyright shall subsist in Canada … in every original literary, dramatic, musical and artistic *work* if any one of the following conditions is met …’ (emphasis added).^44^ Literary work ‘includes tables, computer programs, and compilations of literary works’; artistic work ‘include[s] paintings, drawings, maps, charts, plans, photographs, engravings, sculptures, works of artistic craftsmanship, architectural works and compilations of artistic works’.^45^ Section 5 also establishes a category of ‘work resulting from the selection or arrangement of literary, dramatic, musical or artistic work or of parts thereof, or … the selection or arrangement of data’.^46^ These categories guide the next phase of the analysis, helping determine whether Olivier or Dr Maple have produced a ‘work’.

#### 1. As Applied—Research Participant

One case on point for applying the work requirement to Olivier is *Geophysical Service Inc. v. Encana Corp.*, 2016 ABQB 230 [*GSI*], which illustrates how data recorded from natural phenomena can fit within the definition of ‘artistic or literary’ works.^47^ Although neurodata and seismic data are fundamentally different in the ways they are collected and processed, the core principles of the analysis remain instructive, particularly the observation by the GSI trial court that ‘data becomes a work when it [sic] is compiled’.^48^ The GSI court heard arguments framing the work as artistic and as literary in nature, and considered each in turn.

If Olivier sought to characterize his neurodata as an artistic work, he might rely on GSI’s finding that processed seismic field data constituted an artistic work because ‘the product [was] the result of selection or arrangement of the [raw] data, or sound recordings from the geology of the subsurface’.^49^ The principal difficulty with this approach is that it is only useful for characterizing the outcome neurodata as artistic, not the raw data. Moreover, Olivier himself engaged in no selection or arrangement. It is true that the raw neurodata undergo visual rendering as part of the transformation to outcome neurodata, which may appear to implicate the holding in *DRG Inc v. Datafile Ltd* (1987), 18 CPR (3d) 358 (FCTD), that work expressed in a visual medium is an artistic work.^50^ Here, however, the task of visually representing the data here fell entirely to Dr Maple. Thus, the raw neurodata are unlikely to count as an artistic work, and Olivier is unlikely to be considered an author of the outcome neurodata.

A more promising avenue for characterizing Olivier’s raw dataset as a work can be found in copyright’s conception of a literary work. The threshold for qualifying as a literary work is relatively low: style, merit, and quality are not determining factors.^51^ In *Apple Computer v. Mackintosh Computers Ltd* [1986] 1 F.C. 173 [*Apple*], the courts recognized writings in the form of alphanumeric code as a literary work.^52^ Similarly, Olivier’s responses are captured as code (a binary code matrix rather than alphanumeric lines) and represent a personal expression of his thoughts and reactions to the given scenario, contributing to the creation of the work. This does differ from the writing of computer code featured in *Apple* since Olivier engaged in no effortful translation of intention into an alphanumeric output. The final product, while taking the form of a binary code matrix rather than alphanumeric lines, nonetheless remains a work of written code.

Olivier did not write the code via typing or dictation, but this is not what matters. Indeed, with the development of BCI technologies, it is entirely plausible that a person may soon be able to write computer code akin to that in *Apple* by thought alone. This further collapses any distinctions between Olivier producing neurodata and a coder writing copyright-eligible works of code. One potential remaining difference is that computer programming code is a formal language with explicitly defined syntax and semantics, whereas brain signals can at best be likened to syntax, and their relation to the semantics of thought is a matter of advanced philosophical disputes. Engaging with philosophy of mind debates about functionalism and identity theory would require venturing well beyond the scope of the present discussion. For legal purposes, the analogy between Olivier’s situation and the principles set out in *Apple* is strong enough to serve his argument here since Olivier’s brain activity bears at least some relation to his thoughts and feelings, regardless of which philosophical account one favors.

While terms like ‘literary’ and ‘artistic’ do not ordinarily connote a dataset, copyright law in Canada still recognizes an artistry or literary merit of sorts in code and datasets, as long as they reflect human individuality and intent. Even the naturally occurring seismic signals in *GSI* constituted a literary work, once captured. While no lawyer could fully guarantee Olivier’s success on this prong of the copyright test given how open-textured the key terms are, he nonetheless remains in a defensible position to argue that the raw data reflecting signals from his brain are just as much a work of art or literature as other forms of compiled code or data whose copyright eligibility is undisputed.^53^

#### 2. As Applied—Researcher

Dr Maple’s efforts in producing the processed data likely meet the work requirement. Outcome neurodata in the form of a prediction involves substantial human intervention through interpretation, analysis, and organization. This fulfills the work requirement because the output constitutes a compilation of the various collected data. Albeit with help from the software package and AI, Dr Maple still must arrange information derived from Olivier’s brain and behavior, analyze its significance, and organize the results into a usable form. *GSI* held that processed seismic data satisfied the work requirement because they were compiled, arranged, and fixed in reproducible form.^54^ Similarly, Dr Maple transformed raw data into an organized, interpretable format. Unless this process were to become highly automated with the advance of computational tools, Dr Maple should qualify as having compiled a ‘work’ as understood in copyright law.

### III.E. Originality

While all five elements of the copyright test are analytically important, Canadian copyright experts have noted that ‘[o]riginality is the central requirement of copyright protection’.^55^ The originality requirement stems from the text of the *Copyright Act*, whose protections reach ‘every *original* production in the scientific or artistic domain, whatever may be the mode or form of its expression’ (emphasis added).^56^ The Supreme Court of Canada stated in the landmark case of *CCH Canadian Ltd v. Law Society of Upper Canada*, 2004 SCC 13 [*CCH*], that satisfying the originality requirement demands a non-trivial exercise of skill and judgment; the work must embody something more than ‘a purely mechanical exercise’.^57^ Chief Justice MacLachlan, writing for a unanimous Court, defined skill as ‘the use of one’s knowledge, developed aptitudes or practised ability in producing [a] work’, and judgment as ‘the use of a person’s capacity to discern or form an opinion or evaluation by comparing different possible options in producing [a] work’.^58^

The *GSI* case again provides recent, useful guidance regarding the originality of raw and processed data.^59^ The *GSI* trial court described field seismic data as ‘the original recorded geophysical data, sometimes referred to as basic or raw data, together with the description of the complete recording parameters ... includ[ing] a geophysical shot record, survey information and observers reports’.^60^ The trial court held that ‘the raw or field seismic data is an original literary compilation work and the processed data is both an original literary compilation work and an artistic compilation work in the scientific domain’.^61^ The issue of copyrightability of raw and processed data has not since been tried in Canadian courts. The *GSI* distinction between raw and processed data usefully tracks the contributions of Olivier and Dr Maple.

#### 1. As Applied—Research Participant

*GSI* indicates that raw seismic data constitute an original work, as they are ‘created, not merely collected, through the intervention of human skill’.^62^ This also requires judgment, as data collection involves a multitude of decisions.^63^ These factors impart sufficient originality even though the data are largely derived by gathering signals from inert material, with active human intervention limited to the pulsing of vibrational shots that evoke data for the geophysical shot record. Raw neurodata, being derived from an active and unique human source, exceed this low threshold for originality by a comfortable margin.

We take this view notwithstanding doubts expressed elsewhere. Pull ter Gunne’s writing addresses whether raw neurodata bear ‘the personal stamp of the author’ or embody ‘the result of creative human labor and of creative choices, which are the product of the human spirit’.^64^ She suggests that raw neurodata fall short of these characterizations and therefore lack originality under Dutch law.^65^

Canadian copyright doctrine as developed in *GSI* would likely compel the opposite conclusion: in generating his neurodata, Olivier exercised both skill and judgment. In approaching the Eriksen flanker and go/no-go tasks, he applied his own knowledge, practiced abilities, and learned experiences. He displayed ‘skill and judgment’, his life experiences and cognitive development shaping his approach to the task.^66^ Theo Austin Bruton, writing about the originality requirement as applied to neurodata in the US copyright context, concurs: even if the final output is recorded mechanically, it stems from subjective and necessarily unique mental engagement, therefore bearing hallmarks of originality and an inherently personal nature.^67^

#### 2. As Applied—Researcher

Dr Maple likely satisfies the originality requirement in relation to the outcome data. *GSI* heavily emphasized ‘skill and judgement [being] necessary in the decisions that must be made ... to gather the data’.^68^ The skill and judgment of the researchers in *GSI* closely resembles that of Dr Maple. She had to engage in extensive planning about how to design the study and evoke neural responses that would be useful in neuroprediction, very much akin to the ‘technical planning … to determine how the earth’s floor will be imaged’ involved in seismic data collection.^69^ This far exceeds the low bar set in *CCH*, which asks only for an exercise of skill beyond mere mechanical effort. Furthermore, Dr Maple displayed expertise in complex statistics, neuroscience, and computer science by designing and implementing an ML program that likely meets the minimal standards of skill and judgment articulated in *CCH*, particularly when considered alongside the criteria outlined in the Innovation, Science and Economic Development Canada guidance document.^70^

Moreover, in *GSI*, copyright subsisted in the processed data because the selection and arrangement of the data revealed originality. Dr Maple’s report is original because she used her expertise to determine which brain areas were relevant, interpreted the activity levels, and related the findings to specific questions addressed. In doing so, she negotiated the challenges of research design and data interpretation in a manner distinctive to her and no one else. This is what copyright lawyers mean by originality.

### III.F. Summary

In creating raw and outcome neurodata, Olivier and Dr Maple have unambiguously met some copyright requirements, namely nexus and fixation. Strong arguments support our view that the expression, work, and originality requirements are satisfied as well. While Olivier’s claim appears viable, the work and originality standards would pose the greatest challenges. No doubt, small changes in the study design and methods of the hypothetical case could undercut the strength of the expression and originality arguments, especially as they pertain to Dr Maple and her use of computational tools to automate parts of the data analysis.^71^ It is nonetheless within the reasonable purview of Canada’s courts to recognize copyright in neurodata on the facts under consideration.^72^

Before turning to implications of the analysis, we consider the rights and remedies at stake for the participant and researcher. If granted copyright in the neurodata, both Olivier and Dr Maple would have specific rights for a term of life plus 70 years. The *Copyright Act* affords rightsholders two types of protection: economic rights, which allow creators extensive control over how the work is reproduced and over certain flows of revenue stemming from it; and moral rights, which assign limited, nontransferable protections for the personality and reputation of the author.^73^

Economic rights grant the rightsholder the exclusive permission to make various uses of the work, such as copying, publishing, and adapting.^74^ Such rights can be assigned or licensed to others, allowing for commercial development while enabling creators to benefit financially from their work. For example, Dr Maple’s economic rights flowed in the first instance to her research institution, allowing that employer to license the neurodata to a startup for development of a more advanced recidivism prediction algorithm. Had Olivier not waived them, his economic rights would have allowed him to profit from, or refuse permission for, certain future uses of his neurodata to the extent copyright subsisted in those data.

Moral rights aim to protect the rightsholder’s personality, as the originality of an author’s works stems from ‘unique and intrinsic aspects’ of the self.^75^ Moral rights in Canadian copyright law include attribution, which is the right to be identified as the author of a work.^76^ Another important moral right is that of integrity, which includes ‘the right to object to derogatory treatments or mutilations of the work that would prejudice the author’s reputation’.^77^ Unlike economic rights, moral rights as established under Canada’s *Copyright Act* cannot be transferred. An individual may, however, contractually waive them. Moral rights have grown in significance as technology allows for easier transfer and modification of an author’s work.^78^ Canada’s framework of moral rights is not unique, but the scope and strength of its protections are exemplary among common-law jurisdictions.^79^

Economic and moral rights are separable: the connection between the author’s personality and work persists even if economic rights have been transferred to another party.^80^ While Olivier and Dr Maple have relinquished economic rights in the neurodata, neither one waived their moral rights. Accordingly, each may assert claims pertaining to attribution and integrity. Whether they will prevail in those claims is a question requiring a separate legal analysis, one beyond the scope of this paper. In brief, we note that this analysis would focus on whether the work has been distorted or altered in a manner that harms the author’s reputation.^81^

Consider a scenario where Olivier and Dr Maple have expressed concern about their data supporting predictive models with harmful effects. For example, algorithmic sentencing models may reinforce disparities along racial, gender, or other lines, thereby perpetuating bias against some of Olivier’s fellow inmates who are members of historically marginalized groups, and undermining the integrity of Dr Maple’s research.^82^ A court may decide that this is prejudicial to Olivier’s or Dr Maple’s reputation. Upon finding an infringement of their moral rights, the judge would be free to choose from a full suite of legal and equitable remedies for Olivier or Dr Maple.^83^ One equitable remedy of interest could be a court order compelling the defendant to remove the datasets linked to Olivier or Dr Maple from the neuroprediction algorithm.

## IV. DISCUSSION AND IMPLICATIONS

In the section above, we applied existing legal rules to a fictional scenario in order to better understand how copyright may apply to neurodata in Canada. This exercise revealed a reasonable likelihood that partial copyright protection is available for both Olivier and Dr Maple.

Often, hypothetical exercises like this one serve to furnish a prediction about how courts will decide an issue.^84^ They can also be exercises in persuasion, aimed at establishing that a specified legal outcome is preferable.^85^ Our principal intention, by contrast, is to draw out lessons from the legal analysis that inform further reflections on ethics and policy. Copyright law, as seen above, pays attention to—and at least potentially protects—the human spark embodied in neurodata. In doing so, it sets an example worth following.

Like copyright law, systems of neurodata governance should attend to the skill, judgment, creativity, and human uniqueness on display when individuals like Olivier and Dr Maple undertake the work of neurodata production. The individualized and sensitive nature of neurodata, alongside the potentially regrettable outcomes stemming from their use in criminal risk neuroprediction, shed light on a currently unaddressed need. Whether via copyright law or otherwise, neurodata producers deserve some mechanism for asserting their autonomy or revoking their consent where those data become involved in a harmful use.^86^

We contend that Olivier and Dr Maple have participated in original, expressive human activity by producing neurodata; that activity of this sort engages the core concerns of copyright law; and accordingly, that it would be troubling if these neurodata producers lacked any recourse against future misuse of the data. Highlighting Canada’s moral rights protections, we next offer a policy blueprint for a world currently debating how best to safeguard people’s rights and interests in their brain data.^87^

### IV.A. Political Economy, Data Extractivism, and Labor Justice

As neurotechnology grows more valuable, we expect conversations around neurodata protection to take on greater parallels with data-justice discourse about harvesting data for AI development purposes like the training of large language models. Recent academic literature on data justice sees societal tensions around big data within the context of an extractivist political economy,^88^ with ‘a high pace of “taking” [that] generates benefits for distant capital without generating benefits for local people’.^89^ Commentary in the data justice and data colonialism literature highlights data accumulation by large firms, typically with no compensation or rights afforded to the original producers of data, as a process akin to natural resource extraction.^90^ Because data behave like a valuable commodity whose production usually demands human effort, leading commentators now recommend framing data not as a form of capital, but as a form of labor.^91^ This new approach brings the data justice and labor justice literatures into contact.^92^

These developments intersect with neurodata governance in several ways. For example, neuroethicists have already identified the potential for neurotechnology to aggravate colonial power asymmetries, including through data extraction practices.^93^ The colonial harms of data extraction are magnified in the neuroprediction context, where bias in a recidivism prediction algorithm’s foundational datasets can differentially impact more marginalized groups, thereby perpetuating patterns of social disadvantage.^94^ Alongside these concerns, there are also labor justice considerations running as a common thread through the IP, data justice, and neurodata governance literatures. Copyright law, as seen in the hypothetical case, naturally lends itself to the framing of neurodata as labor: our copyright analysis above repeatedly illustrated how the work of neurodata production captures human effort, skill, creativity, and individuality in a commercially valuable dataset.^95^

This convergence of thematic threads across literatures indicates a need for policy attention to protect the interests of people who produce neurodata. In particular, the negative outcomes identified by literatures on data justice and data colonialism are poised to emerge with respect to neurodata unless countered by copyright or some other policy system. Neurotechnology could soon become the next major theater of the conflict over data subjects’ rights and treatment presently seen in the AI sector.^96^ This is an outcome worth averting. To this end, we perceive a need for focused neuroethics dialogue on the interests of neurodata producers in their capacity as data subjects.^97^

### IV.B. A Moral Rights Approach to Neurodata

The hypothetical case led to our conclusion that Olivier and Dr Maple likely hold moral rights with respect to their neurodata, but have surrendered any economic rights. An outcome like this one leaves individual data subjects with some control over further uses of their neurodata, specifically with respect to attribution and integrity, without assigning full ownership. Whereas comprehensive copyright would grant them expansive power over their data, as moral rightsholders they are afforded more limited abilities. This includes the ability to object to certain harmful misuses of their data.

Not every jurisdiction takes Canada’s robust approach to moral rights, and so not every jurisdiction would realize the benefits of this arrangement by recognizing copyright in neurodata. But any jurisdiction could benefit from protecting neurodata producers in a manner inspired by Canadian moral rights. Indeed, it would be better still if large blocs acted in global coordination—copyright lawyers and neuroethicists alike know the importance of a global framework.^98^ Whether through their judicial systems, administrative agencies, purpose-built legislation, or soft power and nonstate mechanisms, governments serious about protecting data subjects should consider how to provide neurodata producers with what they need: the power to vindicate the unique autonomy, dignity, and privacy interests bound up with their personal data.^99^

Many potential governance models could suffice, including private rights of action enforced via individual litigation, or more pre-emptive regulatory models addressing rights infringement at scale. Moreover, a governance approach framed around moral rights can protect neurodata producers as needed without deciding among competing ownership models for brain data.^100^ Giving people too strong an economic or ownership stake in their neurodata, including through copyright, would upend the political economy of neurotechnology development. It would disrupt settled pathways on a level akin to giving individuals economic rights in subsequent uses of their genetic data, representing a departure from the status quo so costly in political capital that few courts or policymakers could entertain it. A moral rights-inspired approach, affording neurodata producers the means to learn about and resist later misuses of their data, can strike an appropriate balance.^101^

## V. CONCLUSION

Through a hypothetical case study, we have addressed two questions: (i) Are neurodata produced for a neuroprediction study copyrightable under existing Canadian law? (ii) What lessons can this legal analysis offer about the ethics and governance of neurodata? To the first question, we conclude that researchers and participants can plausibly claim copyright in certain neurodata. To the second question, we found compelling reasons to support the creation of a system that protects neurodata producers. By linking these considerations to scholarship on data justice and neurotechnology governance, we underscore the importance of developing fair approaches to ownership and control over neurodata.

We have also offered the preliminary scaffolding for a policy framework that empowers neurodata producers to uphold their own autonomy, privacy, and other interests without owning their data outright. If such a framework does not emerge in Canada, it appears possible that the country’s domestic copyright law could step into the breach, with uncertain results. The task ahead in global neurodata governance is to offer certainty by forging a better path forward.

## Footnotes

1
*See*  Neurotechnologies: The  Next  Technology  Frontier, IEEE Brain, https://brain.ieee.org/topics/neurotechnologies-the-next-technology-frontier/ (last visited June 21, 2025). *See also*  First  Draft of a  Recommendation on the  Ethics of  Neurotechnology (revised version): Working  Document as of 27 August 2024, UNESCO Digital Library (Aug. 27, 2024), https://unesdoc.unesco.org/ark:/48223/pf0000391074.

2 Ari Rotenberg, Kyrstin Lavelle, Reina Magistro Nadler, Margot Gunning, Zelma H.T. Kiss & Judy Illes, *Infringement of Neuromodulation Patents (2000–2024),* __ Neuromodulation: Technology at the  Neural  Interface __ (in press, 2025), at ^*^1.

3 Ari Rotenberg, Stacey Anderson-Redick, Zelma H.T. Kiss, & Judy Illes, *The Neurotechnology Patent Landscape in a Time of Neuroethics*, 12 Human. & Soc. Sci. Communications 633 (2025) at ^*^4.

4
*See* Dylan Roskams-Edris, Stacey Anderson-Redick, Judy Illes, & Zelma H.T. Kiss, *Medical Methods Patents in Neuromodulation*, 22 Neuromodulation: Technology at the  Neural  Interface, 398, 398 (2019); Rotenberg et al., *supra* note 3, at ^*^2; *see also* Rafael Yuste, *Advocating for Neurodata Privacy and Neurotechnology Regulation*, 18 Nature  Protocols 2869, 2869–2870 (2023).

5 Rotenberg et al., *supra* note 3, at ^*^2–3.

6 Dara Hallinan, Philip Schütz, Michael Friedewald, & Paul de Hert, *Neurodata & Neuroprivacy: Data Protection Outdated?* 12 Surveillance & Soc’y 55, 65–69 (2013); *see also* María Isabel Cornejo-Plaza, Roberto Cippitani, & Vincenzo Pasquino, *Chilean Supreme Court Ruling on the Protection of Brain Activity: Neurorights, Personal Data Protection, and Neurodata*, 15 Frontiers in  Psychology 1,330,439 (2024), at ^*^3–4.

7 Hallinan et al., *supra* note 6, at 65.

8
*See* Iris Coates McCall, Chloe Lau, Nicole Minielly, & Judy Illes, *Owning Ethical Innovation: Claims about Commercial Wearable Brain Technologies*, 102 Neuron 728 (2019); *see also* Lauren C. Tindale et al., *Wearable Biosensors in the Workplace: Perceptions and Perspectives*, 4 Front. Digital  Health 800,367 (2022).

9 Hallinan et al., *supra* note 6, at 56; *see also* Stephen Morse, *Neuroprediction: New Technology, Old Problems*, 8:4 Bioethica  Forum 128 (2015).

10 Stephen Rainey et al., *Data and Consent Issues with Neural Recording Devices*, in Clinical  Neurotechnology  Meets  Artificial  Intelligence 241–260 (Oliver Friedrich, Andrea Wolkenstein, Christoph Bublitz, Ralf J. Jox, & Eric Racine, eds., 2021).

11
*See generally* Scott A. Langenecker, Natania A. Crane, Lisanne M. Jenkins, K. Luan Phan & Heide Klumpp, *Pathways to Neuroprediction: Opportunities and Challenges to Prediction of Treatment Response in Depression*, 5 Curr. Behav. Neurosci. Rep. 48 (2018).

12
*See* Eyal Aharoni et al., *Neuroprediction of Future Rearrest*, 110:15 Proc. Nat’l  Acad. Sci. 6223, 6225–27 (2013); *see also* Carl Delfin et al., *Prediction of Recidivism in a Long-term Follow-up of Forensic Psychiatric Patients: Incremental Effects of Neuroimaging Data* 14:5 PLoS One e0217127 (2019) at ^*^3–7; *see also* Kent A. Kiehl et al., *Age of Gray Matters: Neuroprediction of Recidivism* 19 NeuroImage: Clinical 813, 814–18 (2019).

13
*See, eg,* Rainey et al., *supra* note 10; Morse *supra* note 9, at 128; Leda Tortora, Gerben Meynen, Johannes Bijlsma, Enrico Tronci, & Stefano Ferracuti, *Neuroprediction and A.I. in Forensic Psychiatry and Criminal Justice: A Neurolaw Perspective*, 11 Frontiers  Psychol. 220 (2020) at ^*^2.

14 Philipp Kellmeyer, *Big Brain Data: On the Responsible Use of Brain Data from Clinical and Consumer-Directed Neurotechnological Devices*, 14:1 Neuroethics 83 at 85 (2021).

15 Vittorio Stumpo et al., *Machine Learning Algorithms in Neuroimaging: An Overview*, 134 Acta  Neurochirurgica  Supp. 125, 125–28 (2022).

16 Stumpo et al., *supra* note 15, at 125.

17 Tortora et al., *supra* note 13, at ^*^2.

18
*See* Damian O. Eke et al., *International Data Governance for Neuroscience*, 110 Neuron 600 (2022); *see also* Paschal Ochang, Bernd Carsten Stahl, & Damian Eke, *The Ethical and Legal Landscape of Brain Data Governance*, 17 PLoS One e0273473 (2022); B. Tyr Fothergill, William Knight, Bernd Carsten Stahl & Inga Ulnicane*, Responsible Data Governance of Neuroscience Big Data*, 13 Front. Neuroinformatics 28 (2019).

19 Copyright Act, RSC 1985, c. C-42 (Can).

20 Government of Canada, Innovation, *A Guide to Copyright* (10 September 2024), https://ised-isde.canada.ca/site/canadian-intellectual-property-office/en/guide-copyright (accessed Jan. 25, 2025).

21 Daniel J. Gervais, *The Purpose of Copyright Law in Canada*, 2 U. Ottawa L. & Tech. J. 315, 315 (2005). *See also* Théberge v. Galerie d’Art du Petit Champlain Inc.*,* 2002 SCC 34, [2002] 2 S.C.R. 336*;* CCH Canadian Ltd v. Law Society of Upper Canada, 2004 SCC 13, [2004] 1 S.C.R. 339*;* Society of Composers, Authors & Music Publishers of Canada v. Canadian Ass’n of Internet Providers*,* 2004 SCC 45, [2004] 2 S.C.R. 427*.*

22
*See generally*  David Vaver, Intellectual  Property  Law: Copyright, Patents, Trade-Marks*,* 2d ed. (Toronto: Irwin Law 2011). For each of the five elements as set out here, see: Copyright Act, *supra* note 19, at §§ 5(1) & 5(1)(a) (establishing work categories and sufficient nexus conditions); CCH Canadian Ltd v. Law Society of Upper Canada, 2004 SCC 13, [2004] 1 S.C.R. 339, at paras 8 & 14–16 (establishing requirements of originality and expression); Canadian Admiral Corp. v. Rediffusion Inc., [1954] Ex. C.R. 382, 20 C.P.R. 75, at para 28 (establishing the requirement of fixation).

23 Radha M. Pull ter Gunne, *Finding the Flaws in Copyright when Confronted with Neuro-technologies and Brain Data* (LL.M. thesis, Tilburg University, 2018) 1 at 29–34.

24
*See* Aharoni et al., *supra* note 12; Delfin et al., *supra* note 12; Kiehl et al., *supra* note 12; *see also* Josjan Zijlmans et al., *The Predictive Value of Neurobiological Measures for Recidivism in Delinquent Male Young Adults,* 46 J. Psychiatry & Neurosci. E271 (2021) at E271–80.

25 Zijlmans et al., *supra* note 24, at E273.

26
*Id.*

27
*See* Zijlmans et al., *supra* note 24, at E273; *see also* Reshmi Marhe, Ben J.M. van de Wetering & Ingmar H.A. Franken*, Error-Related Brain Activity Predicts Cocaine Use After Treatment at 3-Month Follow-up*, 73 Biol. Psychiatry 782, 783–84 (2013).

28 Aharoni et al., *supra* note 12, at 6225.

29 Théberge v. Galerie d’Art du Petit Champlain inc., 2002 SCC 34, [2002] 2 S.C.R. 336, at para. 30.

30 Copyright Act, *supra* note 19, at § 5(1)(a). Treaty countries also include, eg, Berne Convention jurisdictions.

31 Kenrick v. Lawrence, 25 Q.B.D. 99, 104 (1890).

32 Winkler v. Hendley, 2021 FC 498 at paras 55–59.

33
*See generally* Pull ter Gunne, *supra* note 23.

34 Pull ter Gunne, *supra* note 23, at 56.

35
*See* Copyright Act, *supra* note 19 at §§ 2, 15, 18; Agence du revenu du Québec c. Unidisc Musique Inc., 2021 QCCA 393 (CanLII), paras. 30–36.

36 Andrews v. McHale, 2016 FC 624, at 41.

37 Yuyan Yi et al., *Classification of EEG Signals: An Interpretable Approach Using Functional Data Analysis*, 376 J. Neurosci. Methods 109,609 (2022) at ^*^1.

38
*See Andrews, supra* note 36, at 88 (from which we have drawn the above-quoted ‘intellectual effort’ phrasing).

39 We allow for the possibility that some forms of functional neurodata may pose closer questions for the expression analysis—for example, the activity of the default mode network. *See generally* Vinod Menon, *20 Years of the Default Mode Network: A Review and Synthesis*, 111 Neuron 2469 (2023).

40 Kenrick, *supra* note 31; Winkler, *supra* note 32.

41 It is true that Dr Maple used a software package and machine-learning algorithmic tools, which partially automated the work of arranging data and drawing inferences from known variables. This slightly reduces the level of human interpretation and creative presentation involved, but on the facts as described, Dr Maple has still done more than enough to meet the expression requirement.

42 Canadian Admiral Corp. v. Rediffusion Inc., [1954] Ex. C.R. 382, 20 C.P.R. 75, at 402.

43
*See* Canadian Intellectual Property Office, *Copyright – Learn the Basics, available at*  https://ised-isde.canada.ca/site/canadian-intellectual-property-office/en/copyright-learn-basics/copyright-learn-basics-protect-your-original-works-learn-why-copyright-matters (accessed Jan. 6, 2026); *accord* Entertainment Software Ass’n v. Society of Composers, Authors and Music Publishers of Canada, 2012 SCC 34, at para 5.

44 Copyright Act, RSC 1985, c. C-42, § 5 (Can). Sections 15, 18, and 21 of the Act establish other copyrightable subject-matter, including performances, sound recordings, and communication signals. Our references to a ‘work requirement’ should therefore be understood as shorthand for a requirement that a copyrightable product must either be a work *or* some other form of copyrightable subject matter. Since the only category relevant here is a (literary, dramatic, musical, or artistic) work, we have omitted further reference to other categories.

45
*Id*.

46
*Id*.

47 Geophysical Service Inc. v. Encana Corp., 2016 ABQB 230, at paras 75–78; Geophysical Service Inc. v. Encana Corp., 2017 ABCA 125.

48 GSI (ABQB), *supra* note 47, at para 77.

49
*Id*. at 76.

50 DRG Inc. v. Datafile Ltd, 18 C.P.R. (3d) 358 (F.C.T.D. 1987) at para 15.

51 University of London Press, Ltd v. University Tutorial Press, Ltd, [1916] 2 Ch. 601, 608 (Chancery Division, 1916).

52 Apple Computer Inc. v. Mackintosh Computers Ltd, [1986] 1 F.C. 173 at paras 50–52.

53 In arguing this point, Olivier would also need to explain why the expression analysis here should not be governed by Glen Gould Estate v. Stoddart Publishing Co. Ltd, 1988 CanLII 5513 (ON CA). That case held that copyright does not subsist in the unfixed oral utterances of an interviewee, only in the notes of those utterances taken by the interviewer. Opposing counsel would seek to liken Olivier to the interviewee in *Gould Estate*, but Olivier is in fact more similarly positioned to the earth in GSI, as the originator of raw data that indeed constituted a copyright-eligible literary work. While *Gould* may represent a somewhat adverse precedent, it is an older case whose analysis stands in tension with the more applicable and recent reasoning of *GSI* and *Apple*, while also being distinguishable on its facts.

54 GSI (ABQB), *supra* note 47, at paras 74–77.

55 Carys J. Craig, *The Evolution of Originality in Canadian Copyright Law: Authorship, Reward and the Public Interest*, 2 U. Ottawa L. & Tech. J. 425, 425 (2005).

56 Copyright Act, *supra* note 19, at § 2.

57 CCH Canadian Ltd v. Law Society of Upper Canada, 2004 SCC 13, [2004] 1 S.C.R. 339 at para 16.

58
*Id.* at 16.

59 GSI (ABQB), *supra* note 47, at paras 78–85.

60
*Id.* at para 47.

61 GSI (ABQB), *supra* note 47, at para 115. Subsequent procedural history indicates that this is likely a general rule beyond Alberta. The court of appeal upheld this decision, observing that the trial judge made no errors in law. The Supreme Court of Canada later dismissed an application for leave to appeal the judgement (Geophysical Services Incorporated v. Encana Corporation*,* 2017 SCC 37634).

62
*Id*. at paras 79–82.

63
*Id.*

64 Pull ter Gunne, *supra* note 23, at 43.

65
*Id*.

66 Venkat Ram Reddy Ganuthula, *The Limits of Personal Experience*, 15 Front. Psychol. 1365180, 1 at 1 (2024).

67 Theo Austin Bruton*, Mind-Movies: Original Authorship as Applied to Works From ‘Mind-Reading’ Neurotechnology*, 14 Chi.-Kent J. Intell. Prop. 263, 277–86 (2014).

68 GSI (ABQB), *supra* note 47, at para 56.

69 GSI (ABQB), *supra* note 47, at para 50.

70
*See* Craig, *supra* note 57. *See also* Innovation, Science and Economic Development Canada, A Consultation on a Modern Copyright Framework for Artificial Intelligence and the Internet of Things, *available at*  https://ised-isde.canada.ca/site/strategic-policy-sector/en/marketplace-framework-policy/copyright-policy/consultation-modern-copyright-framework-artificial-intelligence-and-internet-things-0 (accessed Jul. 25, 2025).

71 Considering the broad term ‘neurodata’ and the technologies associated with it—eg neuromodulation devices or neuroprosthetic brain–computer interfaces—there could indeed be as many variations in the copyright analyses of different cases as there are different methods for capturing and processing brain data. We have deliberately limited the scope of our analysis to the facts at hand because the key conclusion is simply that copyright may situationally subsist in at least *some* varieties of neurodata, with attendant ethical implications.

72 Encouragingly, Bruton and Pull ter Gunne reach similar conclusions when addressing this question in their own jurisdictions. We note also the existence of a more mixed, partly contrary view offered in Jonathan Baker, *The Advent of Effortless Expression: An Examination of the Copyrightability of BCI-Encoded Brain Signals*, 105 Minn. L. Rev. 389 (2020). Baker’s normative contention is that neurodata should not enjoy copyright eligibility because utilitarian copyright theory counsels against it; however, his doctrinal analysis points to the same positive-law conclusion we reach, that neurodata likely satisfy the extant legal test for copyright. *Id*. at 398–406.

73 Eugene C. Lim, *On the Uneasy Interface between Economic Rights, Moral Rights and Users’ Rights in Copyright Law: Can Canada Learn from the UK Experience,* 15 SCRIPTed 70, 72–76 (2018).

74 Margaret Ann Wilkinson & Natasha Gerolami, *The Author as Agent of Information Policy: The Relationship Between Economic and Moral Rights in Copyright*, 26 Gov’t  Info. Q. 321, 332 (2009).

75 Emir Aly Crowne Mohammed, *Moral Rights and Mortal Rights in Canada*, 4 J. Intell. Prop. L. & Prac. 261, 261 (2009).

76 Lim, *supra* note 73, at 75.

77
*Id.*

78 Wilkinson & Gerolami, *supra* note 74.

79
*See* Elizabeth Adeney, *Moral Rights in Canada: an Historical and Comparative View*, in An  Emerging  Intellectual  Property  Paradigm: Perspectives from  Canada*,* 163, 195–96 (Ysolde Gendreau ed., 2008) & Mira T. Sundara Rajan, *Culture Matters: Why Canada’s Proposed Amendments to Its Copyright Law Should Revisit Moral Rights*, in From ‘Radical  Extremism’ to ‘Balanced  Copyright’: Canadian  Copyright and the  Digital  Agenda 476, 486 (Michael Geist ed., 2010). *See generally*  Mira T. Sundara  Rajan, Moral  Rights: Principles, Practice and  New  Technology (2010).

80 Lim*, supra* note 73 at 78; Mira T. Sundara Rajan, *The Moral Rights of Authors and Artists: From the Birth of Copyright to the Age of Artificial Intelligence* (Oxford University Press 2025) at 37–38.

81 Copyright Act, *supra* note 19, at § 28.22. Promisingly, part of the legal test determining infringements of the right to integrity focuses on whether the work is used ‘in association with a product, service, cause or institution’ in a way that prejudices the author’s honour or reputation.

82 This concern applies equally to neuropredictive and conventional risk assessment methods. *See* Roland Neil & Michael Zanger-Tishler, *Algorithmic Bias in Criminal Risk Assessment: The Consequences of Racial Differences in Arrest as a Measure of Crime*, 8 Ann. Rev. Criminol. 97 (2025) & Tim O’Brien, *Compounding Injustice: the Cascading Effect of Algorithmic Bias in Risk Assessments*, 13:39 Geo. J. L. Mod. Crit. Race  Persp. 39 (2021).

83
*Id.*

84 While we discern a viable path for Canadian courts to recognize copyright in neurodata, we refrain from predicting this outright. The common-law tests for the copyright factors continually evolve, influenced by considerations of administrability, fairness, and deference to Parliament. Courts might decline to extend protection here because doing so could disrupt conventional pathways in research and technology development.

85 Although there are reasons to prefer legal recognition of copyright in neurodata, we have taken care to avoid suggesting that this result would be politically or economically optimal overall. The ramifications of such a development in copyright law are unknown and remain a pressing question for further research. Our normative contentions in this article are about what neurodata producers are owed, not about which system owes it to them.

86 A growing body of scholarship understands copyright as protective of autonomy. *See, eg,* Christopher S. Yoo, *Rethinking Copyright and Autonomy*, 2019(3) U Ill. L. Rev 1039 (2019); Shyamkrishna Balganesh, *Privative Copyright*, 73 Vand L. Rev 1 (2020); Peter Lee, *Autonomy and Structures of Creative Production*, 83:2 Ohio  St. LJ 283 (2022). Our approach is complementary to these, differing slightly in that we emphasize the potential to vindicate an autonomy interest in personal data via assertions of moral rights.

87
*See, eg,* Eke et al., *supra* note 18; Marcello Ienca et al., *Towards a Governance Framework for Brain Data*, 15 Neuroethics 20 (2022); Erica Harper & Timo Istace, *Neurotechnology and Human Rights: An Audit of Risks, Regulatory Challenges, and Opportunities* (Geneva Academy of International Humanitarian Law and Human Rights, Mar. 27, 2025); Ricardo Chavarriaga, Jean-Marc Rickli, & Federico Mantellassi, *Neurotechnologies: The New Frontier for International Governance* (Geneva Centre for Security Policy, Apr. 2023).

88
*See, eg,* David Thatcher, David O’Sullivan, & Dillin Mahmoudi, *Data Colonialism Through Accumulation by Dispossession: New Metaphors for Daily Data*, 34(6) Env’t & Planning D: Soc’y & Space 990 (2016); Nick Couldry & Ulises A. Meijas, *Making Data Colonialism Liveable: How Might Data’s Social Order Be Regulated?* 8(2) Internet  Pol’y  Rev. 1 (2019). *See also* Linnet Taylor, *What Is Data Justice? The Case for Connecting Digital Rights and Freedoms Globally*, 2017 Big  Data & Soc’y 1 (2017) (on the meaning of data justice generally).

89 Dayna Nadine Scott, *Extractivism: Socio-legal Approaches to Relations with Lands and Resources*, in The  Routledge  Handbook of  Law & Society 124 (Mariana Valverde, Kamari M. Clarke, Eve Darian Smith, & Prabha Kotiswaran eds., 2021). The copyright system itself is implicated in critical discussions of big-data extractivism. *See* Daryl Lim, *Banana Republic: Copyright Law and the Extractive Logic of Generative AI*, 2025 J. Intell  Prop. L. & Prac. __ (online first, 2025). Like other commentators concerned about the extractive dynamics of copyright, we envision a system purpose-built to promote social and economic justice for data subjects. *See, eg,* Latif Mtima, *Copyright Social Utility and Social Justice Interdependence: A Paradigm for Intellectual Property Empowerment and Digital Entrepreneurship*, 112 W. Va. L. Rev. 97 (2009); Lateef Mtima, *Copyright and Social Justice in the Digital Information Society: ‘Three Steps’ Toward Intellectual Property Social Justice*, 53:2 Houst. L. Rev. 459 (2015); Steven D. Jamar, *A Social Justice Perspective on the Role of Copyright in Realizing International Human Rights*, 25 Pac. McGeorge  Global  Bus. & Dev’t LJ 289 (2012).

90
*See* Payal Arora, *Creative Data Justice: a Decolonial and Indigenous Framework to Assess Creativity and Artificial Intelligence*, 2024 Information, Communication & Soc’y 557 (2024). *See also* Spyros Spyrou, *From Extractivist Practices and the Child-as-data to an Ethics of Reciprocity and Mutuality in Empirical Childhood Research*, 31 Childhood 3 (2024) (‘In line with its extractivist logic, data is often metaphorically talked about as a natural resource, much like any other natural resource which can be extracted and used for economic profit or for personal gain’.)

91
*See* Imanol Arrieta Ibarra, Leonard Goff, Diego Jiménez-Hernández, Jaron Lanier, & E. Glen Weyl, *Should We Treat Data as Labour? Moving Beyond ‘Free’* 108 AEA Papers & Procs. 38 (2018); Julian David Jonker, *Is Data Labor? Two Conceptions of Work and the User-Platform Relationship*, 35(2) Bus. Ethics  Quarterly 153.

92 Scholars have thus proposed a labor/data justice framework. *See* Deirdre McCann & Arely Cruz-Santiago, *Labour/Data Justice: A New Framework for Labour/Regulatory Datafication*, 49 J. L. & Soc’y 658 (2022). Copyright scholars, too, have connected data justice and labor justice. *See* Kalpana Tyagi, *Copyright, Text & Data Mining and the Innovation Dimension of Generative AI*, 19:7 J. Intell. Prop. L. & Prac. __ (online first, 2024).

93
*See* Judy Illes, Makarena Dudley, Lucia Machova Urdzikova, Ioana Podina, & Monique Pyrrho, *The Risk of Neurotechnology as an Instrument of Colonialism*, 7(3) Brain  Comms. 1 (2025).

94
*See* Tortora et al., *supra* note 13, at ^*^4–5; *see also* Jingwei Li et al., *Cross-ethnicity/race Generalization Failure of Behavioural Prediction from Resting-state Functional Connectivity*, 8:11 Sci. Advances eabj1812 (2022) & Rongguang Wang, Pratik Chaudhari, & Christos Davatzikos, *Bias in Machine Learning Models Can Be Significantly Mitigated by Careful Training: Evidence from Neuroimaging Studies*, 120:6 Proc. Nat’l  Acad. Sci. USA e2211613120 (2023). The use of neuroprediction in colonial legal systems further risks imposing a neuroreductionistic worldview on Indigenous populations in a culturally inappropriate manner. *See* Illes et al., *supra* note 93, at 1 (describing the worldview reflected in rationalist technoscientific projects as historically functioning to ‘delegitimize alternative ways of living, knowing, and thinking … [thereby] marginalizing Indigenous epistemologies and overlooking their sustainable approaches to health, well-being and the environment’.).

95 Recent scholarship has explicitly linked copyright law and labor policy, which collide in high-profile disputes about data extraction and creative labor relations. *See* Xiyin Tang, *Intellectual Property Law as Labor Policy*, 100 NYU L. Rev 62 (2025) (framing copyright law as a forum for labor conflict in the sense that employers and employees in the creative labor sector have adverse interests over copyright).

96
*See* Will Douglas Heaven, *What Comes Next for AI Copyright Lawsuits?* MIT Tech. Rev. (Jul. 1, 2025) (*available at*  https://www.technologyreview.com/2025/07/01/1119486/ai-copyright-meta-anthropic/).

97 Such a dialogue would run parallel to discussions in the biomedical and health literatures about involving data subjects in governance. *See* Effy Vayena & Alessandro Blasimme, *Biomedical Big Data: New Models of Control Over Access, Use and Governance*, 14 Bioethical  Inquiry 501, 503 (2017).

98
*See, eg,* Marcello Ienca et al., *Towards a Governance Framework for Brain Data*, 15 Neuroethics 20 (2022); Damian O. Eke et al., *International Data Governance for Neuroscience*, 110 Neuron 600 (2022); B. Tyr Fothergill, William Knight, Bernd Carsten Stahl & Inga Ulnicane*, Responsible Data Governance of Neuroscience Big Data*, 13 Front. Neuroinformatics 28 (2019).

99 Cf. Vayena & Blassime, *supra* note 82, at 501 (‘The capacity to exert control over the generation and flow of personal information is a fundamental premise to important values such as autonomy, privacy, and trust’.)

100
*Cf.* Jakub Piasecki & Phaik Yeong Cheah, *Ownership of Individual-Level Health Data, Data Sharing, and Data Governance*, 23 BMC Med. Ethics 104 (2022) (comparing privatization and communization as ownership models in the related context of health data). *See also* Mark A. Hall*, Property, Privacy, and the Pursuit of Interconnected Medical Records*, 95 Iowa L. Rev. 631 (2010).

101 A proposal similar to ours can be found in the AI governance space. It seeks to ‘facilitate a system of rapid notice of potentially harmful [AI] applications and objects’. Cason D. Schmit, Megan J. Doerr, & Jennifer K. Wagner, *Leveraging Copyright for AI Governance*, 379 Science 646, 646 (2023). Rather than centralized enforcement, however, we envision neurodata producers taking action in groups or individually where subsequent uses of their neurodata appear harmful or prejudicial.

